# Multiplexed Reverse Transcription Loop-Mediated Isothermal Amplification Coupled with a Nucleic Acid-Based Lateral Flow Dipstick as a Rapid Diagnostic Method to Detect SARS-CoV-2

**DOI:** 10.3390/microorganisms11051233

**Published:** 2023-05-07

**Authors:** Derich Shalbie Simon, Chee-Wei Yew, Vijay Subbiah Kumar

**Affiliations:** Biotechnology Research Institute, Universiti Malaysia Sabah, Kota Kinabalu 88400, Malaysia; derich_shalbie_simon_mz20@iluv.ums.edu.my (D.S.S.); cheewei.yew@ums.edu.my (C.-W.Y.)

**Keywords:** SARS-CoV-2, COVID-19, diagnosis, loop-mediated isothermal amplification, lateral flow dipstick, nucleocapsid gene, membrane gene, envelope gene

## Abstract

Due to the high reproduction rate of COVID-19, it is important to identify and isolate infected patients at the early stages of infection. The limitations of current diagnostic methods are speed, cost, and accuracy. Furthermore, new viral variants have emerged with higher rates of infectivity and mortality, many with mutations at various primer binding sites, which may evade detection via conventional PCR kits. Therefore, a rapid method that is sensitive, specific, and cost-effective is needed for a point-of-care molecular test. Accordingly, we developed a rapid molecular SARS-CoV-2 detection kit with high specificity and sensitivity, RT-PCR, taking advantage of the loop-mediated isothermal amplification (LAMP) technique. Four sets of six primers were designed based on conserved regions of the SARS-CoV-2 genome: two outer, two inner and two loop primers. Using the optimized protocol, SARS-CoV-2 genes were detected as quickly as 10 min but were most sensitive at 30 min, detecting as little as 100 copies of template DNA. We then coupled the RT-LAMP with a lateral flow dipstick (LFD) for multiplex detection. The LFD could detect two genic amplifications on a single strip, making it suitable for multiplexed detection. The development of a multiplexed RT-LAMP-LFD reaction on crude VTM samples would be suitable for the point-of-care diagnosis of COVID-19 in diagnostic laboratories as well as in private homes.

## 1. Introduction

Severe acute respiratory syndrome coronavirus 2 (SARS-CoV-2) was first identified in December 2019 during an outbreak originating from a market in Wuhan, China [1]. This novel coronavirus is the cause of coronavirus disease 2019 (COVID-19) and is only the seventh known coronavirus that has been infectious to humans [2,3]. The rapid spread of COVID-19 has resulted in the World Health Organization (WHO) declaring a global pandemic. The rising number of infected individuals has caused the collapse of hospital systems as they cannot be accommodated [4,5]. This led to countries deciding to close their borders to manage the spread of the disease. However, with many countries adopting national vaccination policies, international travel opened again. Therefore, variants from all around the world were allowed to spread worldwide [6,7]. However, due to the rapidly mutating nature of the viral RNA, many new variants emerged with the ability to evade vaccine-induced immunity. Mutations on common PCR primer binding sites cause the misdiagnosis of COVID-19 via RT-PCR tests [8].

The spread of viral disease can be controlled by quickly identifying and isolating infected individuals. Therefore, it is important to have rapid and accurate diagnostic assays. Throughout the pandemic, various techniques were described and employed for the mass screening of COVID-19. This included real-time reverse transcription polymerase chain reaction (real-time RT-PCR) detection, droplet digital PCR (ddPCR) [9,10], Clustered Regularly Interspaced Palindromic Repeats (CRISPR) [11], nanomaterial-based techniques [12], antigen rapid tests (RTK-Ag), antibody rapid tests (RTK-Ab), cell culture, electron microscopy to the chest and CT-scans [13]. However, these techniques were limited by the lack of facilities, trained personnel, and accuracy.

Currently, the common methods used are RT-PCR detection and RTK-Ag. RTK-Ag is widely used for its rapidness and ease of use but is not as accurate as RT-PCR. RTK-Ag detects the viral proteins that are already present in the patient samples. Meanwhile, RT-PCR is regarded as the gold standard in SARS-CoV-2 detection as it can amplify low amounts of viral genetic material to a detectable amount [14,15]. Therefore, this test is highly specific and sensitive but requires trained personnel and advanced facilities, is expensive and requires a long time. As an alternative, isothermal nucleic acid amplification methods such as Loop-mediated isothermal amplification (LAMP) have been established as a rapid, specific, sensitive, and robust diagnostic method suitable for high-throughput screening [16,17].

LAMP is a method to exponentially amplify a specific nucleic acid region at isothermal conditions [18,19]. Amplification can be observed within 15–60 min at 60 °C to 65 °C. The target regions can be amplified with high efficiency by only using a heating block or water bath, solving the temperature dependency of PCR. Therefore, this method does not require expensive equipment such as a thermocycler or real-time PCR machine and can be performed by individuals without prior training. This method is suitable for clinical diagnostics in a resource-poor environment. LAMP (and RT-LAMP) are commonly used in diagnostic microbiological fields to detect pathogens such as viruses (HIV [20], SARS-CoV-1 [21] and MERS-CoV [22]), bacteria (Tuberculosis [23] and *Salmonella* [24]), nosocomial bacteria (*Acinetobacter baumannii*) [25], fungal pathogens (*Pneumocystis jirovecii*) [26] and parasites (*Ortleppascaris sinensis* [27] and *Phytophthora ramorum* [28]), as well as in the detection of antibiotic-resistant genes (β-lactamases genes [29]).

LAMP products can be visualized either by agarose electrophoresis or through colorimetry. These visualization techniques come with their own advantages and disadvantages. However, in a multiplexed system, these methods cannot be used to differentiate the amplified targets. Therefore, the use of a Lateral Flow Dipstick (LFD) is best suited to detect and differentiate target genes when performing multiplex amplifications. Here, each set of primers could be modified with specific antigen labels to enable rapid detection with the LFD.

In this study, novel LAMP primers were designed to detect SARS-CoV-2 Nucleocapsid (N), Membrane (M) and Envelope (E) genes. These primers are designed on conserved regions of the SARS-CoV-2 genes and aligned against closely related coronaviruses. Using a strand displacing DNA polymerase with reverse transcription activity, a single-enzyme RT-LAMP reaction was achieved. This reaction could detect 100 copies of the control plasmids in 30 min, which could be observed through the formation of bands on a lateral flow dipstick or color changes by SYBR Green Staining. We believe this method will be useful as an alternative to current techniques and helpful as a resource in poorer countries for the rapid diagnosis of the virus.

## 2. Materials and Methods

### 2.1. Primer Design for PCR and LAMP Assays

The published sequence from Genbank (Accession number NC_045512.2) was used as the reference sequence for the primer design. In addition, full genome sequences of SARS-CoV-2 were collected from GISAID (Appendix A Table A1), and the N, M and E gene regions were identified. Multiple sequence alignment was conducted on each gene to identify the conserved regions of each gene. These regions were used as inputs in PrimerExplorer version5 http://primerexplorer.jp/lampv5e/index.html (accessed on 3 January 2021) to obtain the F1, B1, F2, B2, F3 and B3 sites for the LAMP primer design. Once desired regions were selected, loop primers were then generated using the same software.

For each gene, a set of primers was designed, consisting of two inner primers (FIP and BIP), two outer primers (F3 and B3) and two loop primers (LF and LB). The forward inner primer (FIP) was designed by a combination of the complementary sequence of F1 (F1c) and F2, linked by a poly-T linker. Additionally, and similarly, the backward inner primer (BIP) was a combination of B1c and B2 with a poly-T linker as well (Table 1).

### 2.2. Preparation of DNA Template

The target N, M and E genic regions were amplified via PCR using the outer primers of each primer set. The PCR reactions were as described [30] with modifications. The reaction of 25 µL consisted of a 1X PCR buffer, 1.5 mM MgCl_2_, 0.2 mM dNTP mix (Promega, Madison, WI, USA), 20 pmol of each outer primer and 0.2 U *Taq* DNA polymerase (Promega, Madison, WI, USA). The thermocycler protocol included an initial denaturation of 95 °C for 5 min, followed by 40 amplification cycles of 95 °C for 30 s, 55 °C for 30 s and 72 °C for 30 s, and a final extension at 72 °C for 10 min. PCR products were viewed on agarose gel, which were then excised and purified using a QIAquick Gel Extraction Kit (Qiagen, Germantown, MD, USA).

### 2.3. Recombinant Plasmid Construction for Positive Control for PCR and LAMP Analysis

The purified PCR products were each ligated into a pJET.2 Blunt Cloning Vector in accordance with the CloneJET Blunt End PCR Cloning Kit (Thermo Fisher Scientific, Waltham, MA, USA) instructions. The recombinant plasmids were transformed into 50 µL of a chemically competent *E. coli* strain TOP10, which were then cultured on an ampicillin-Luria Bertani (LB) agar plate at 37 °C for 16 h. Single colonies were selected and grown in an LB broth at 37 °C for 16 h, followed by plasmid extraction using a GeneJET Plasmid Miniprep Kit (Thermo Fisher Scientific, USA) according to the manufacturer’s protocol. The plasmid was then verified through DNA sequencing.

### 2.4. RNA Synthesis for RT-LAMP Protocol Verification

Synthetic RNA for each gene was synthesized from their respective plasmid using a HiScribe ^®^ T7 Quick High Yield RNA Synthesis Kit (New England Biolab, Hitchin, UK). The protocol was conducted as per the manufacturer’s instructions. Synthetic RNA was purified using the lithium chloride protocol [31].

### 2.5. Optimization of LAMP and RT-LAMP Reaction Condition with UV Analysis

Initially, the LAMP assay was conducted modified based on a previously described protocol with a 30 µL reaction mixture containing a 1X Isothermal Amplification Buffer II (New England Biolab, UK), 0.4 M of Betaine (Sigma-Aldrich, St. Louis, MO, USA), 8 mM of MgSO_4_, a 1.4 mM dNTP mix (Promega, USA), 10 U *Bst* of 3.0 DNA polymerase (New England Biolab, UK), 32 pmol of each inner primer, 8 pmol of each outer primer, 32 pmol of each loop primer, followed by 2 µL of template DNA or RNA [32]. Optimizations were performed by testing different ratios of outer, inner and loop primers, as well as the working concentrations of MgSO_4_.

### 2.6. Sensitivity Test for the Detection of SARS-CoV-2 Nucleocapsid, Envelope and Membrane Genes Using End Point-PCR and Quantitative PCR

The recombinant plasmid DNA with each gene was serially diluted 10-fold to achieve 10^8^ copies to one copy number [30]. Both the endpoint and quantitative PCR were conducted on each dilution of recombinant plasmid using the outer primers stated in Table 1. For the endpoint PCR, the protocol was as mentioned in Section 2.2. Subsequently, the amplification products were visualized on 2.0% agarose gel electrophoresis, stained with ethidium bromide, and observed under UV light. In addition, quantitative PCR (qPCR) was conducted with the addition of an SYBR Green stain using a real-time PCR machine (Biorad CFX96, Hercules, CA, USA).

### 2.7. Sensitivity Test for the Detection of SARS-CoV-2 Nucleocapsid, Envelope and Membrane Genes Using LAMP-UV, LAMP-SYBR Green and LAMP-LFD Analyses

The optimized LAMP protocol was conducted on the same set of 10-fold serial dilution positive control recombinant plasmids for 30 min at 65 °C UV, and SYBR Green and LFD analyses were used to visualize the amplification products of the LAMP assays. The 1.5% agarose gel electrophoresis was conducted on amplification products, stained in ethidium bromide and observed under UV conditions. Colorimetry using SYBR Green was conducted by the addition of 2 µL of 1:10, which was diluted SYBR Green I nucleic acid gel stain to all tubes containing LAMP products, and observations on the color changes were immediate [30]. As for LFD, the LAMP protocol was conducted using the primers stated in Table 1, where inner primers were labeled with specific antigens for LFD detection. The PCRD Flex Nucleic Acid-Based Immunoassay (Abingdon Health, York, UK) was used according to the manufacturer’s instructions.

### 2.8. Specificity Test of LAMP Assay

The specificity of each set of LAMP primers was evaluated in silico and in vitro. The sequences of closely related coronavirus, regardless of the hosts, were downloaded from Genbank (AY613950.1, KY352407.1, KY417144.1, NC_001451.1, NC_002645.1, NC_004718.3, NC_005831.2, NC_006213.1, NC_006577.2, NC_038294.1, NC_048213.1) and aligned on MEGA X [33]. Mismatches were observed on sequences of the primer binding regions on viral sequences that did not belong to SARS-CoV-2. For comparison, the synthetic DNA of SARS-CoV-1 and MERS-CoV genes, as well as the cDNA of Infectious Bronchitis Virus (IBV), were used for in vitro specificity tests as these were the ones available. The LAMP assays were performed at 65 °C for 30 min with 20 ng of each control.

## 3. Results

### 3.1. LAMP and RT-LAMP Optimization Using UV Analyses

**Figure 1:**
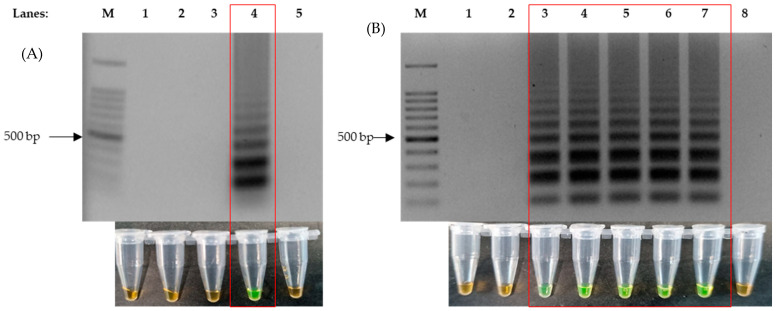
Formation of ladder-like bands on agarose gel electrophoresis produced by LAMP reaction and colorimetric changes of SYBR Green from orange to green, as highlighted in the red box. (**A**) The correct ratio of inner, outer and loop primers for successful LAMP reaction (LAMP-UV, top and LAMP-SYBR Green, bottom), lane 1: 100 bp DNA ladder, lane 2: primer ratio of 1:1:1, lane 3: primer ratio of 2:1:2, lane 4: primer ratio of 4:1:4 and lane 5: primer ratio of 8:1:8. (**B**) Agarose gel electrophoresis results of 8 mM of added MgSO_4_ against time (LAMP-UV, top and LAMP-SYBR Green, bottom), lane M: 100 bp ladder, lane 1: 1 min, lane 2: 5 min, lane 3: 10 min, lane 4: 15 min, lane 5: 20 min, lane 6: 25 min, lane 7: 30 min and lane 8: non-template control at 30 min.

The optimization of the LAMP protocol was performed with the purpose of shortening the time required for amplification without compromising on sensitivity. Positive LAMP reactions were indicated by the formation of ladder-like bands after performing agarose gel electrophoresis, stained with ethidium bromide, and these were observed under UV light. Four ratios of Outer:Inner:Loop primers were used, 1:1:1, 2:1:2, 4:1:4 and 8:1:8, to perform the LAMP assay on the recombinant plasmid DNA. Thus, we observed that a 4:1:4 primer ratio produced DNA amplification with every set of primers (Figure 1A). Subsequent to identifying the right ratio of the primers in use, the same protocol was repeated against synthetic RNA to test for the single enzyme one-step RT-LAMP protocol. To further optimize the reaction time, 4 mM, 6 mM, 8 mM and 10 mM of MgSO_4_ was used for the assay and tested on an incubation time of 5 min intervals (5, 10, 15, 20, 25 and 30), which showed that 8 mM had the fastest reaction speed; this allowed for amplification without having amplifications at the non-template control (NTC). The results showed that the addition of 8 mM of MgSO_4_ was able to produce an amplification within 10 min (Figure 1B). This was also the fastest reaction speed in comparison to the other MgSO_4_ concentrations.

### 3.2. Sensitivity Test of End Point-PCR, Quantitative PCR, LAMP-UV and LAMP-SYBR for the Detection of SARS-CoV-2 Genes

The sensitivity test was successfully conducted using a set of SARS-CoV-2-positive control plasmids which were serially diluted 10-fold, from 10^8^ copies to just one copy. The visualization methods of the PCR (end-point and quantitative) and LAMP (UV, SYBR Green and LFD) were observed to not have had any effect on the sensitivity. The sensitivity of PCR and the optimized LAMP protocol varied between the different target gene types. However, ultimately, the optimized LAMP protocol proved to be equally sensitive and, on certain genes, more sensitive when compared to the PCR.

**Figure 2:**
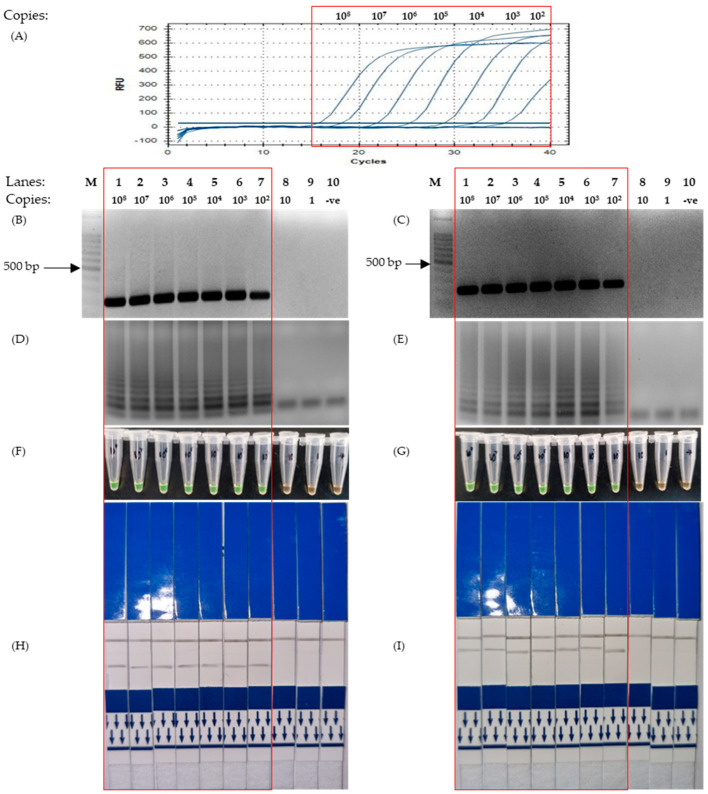
Results for sensitivity test of LAMP using primers designed based on the Nucleocapsid gene, conducted using a serial-diluted positive control plasmid from 100,000,000 to a single copy. The red box highlights the positive detection of SARS-CoV-2 N genic regions. (**A**) Quantitative Polymerase Chain Reaction using the protocol mentioned in 2.6. (**B**,**D,F**,**H**) Corresponding results of End-point PCR, LAMP-UV, LAMP-SYBR Green and LFD, respectively, using primers targeting the N1 region of the Nucleocapsid gene. (**C**,**E**,**G**,**I**) Corresponding results of End-point PCR, LAMP-UV, LAMP-SYBR Green and LFD, respectively, using primers targeting the N2 region of the Nucleocapsid gene. Lane M: 100 bp ladder, lane 1: 10^8^ copies, lane 2: 10^7^ copies, lane 3: 10^6^ copies, lane 4: 10^5^ copies, lane 5: 10^4^ copies, lane 6: 10^3^ copies, lane 7: 10^2^ copies, lane 8: 10 copies, lane 9: 1 copy, lane 10: non-template control.

For the detection of the nucleocapsid (N) gene, two sets of LAMP primers were designed to target two different regions, namely N1 and N2. However, following the sensitivity test, it was revealed that the sensitivity of both primers set across all assays was identical. As revealed in Figure 2A,B, the detection limit for both end-points and quantitative PCR was 10^2^ copies of plasmids using an N1 primer set, with the corresponding LAMP-UV, LAMP-SYBR Green, and LAMP-LFD assays providing a detection limit of 10^2^ copies as well (Figure 2D,F,H). Conversely, for the N2 primer set, the results showed that the detection limit across all assays (quantitative PCR, end-point PCR, LAMP-UV, LAMP-SYBR Green and LAMP-LFD) was 10^2^ copies, respectively (Figure 2A,C,E,G,I).

**Figure 3:**
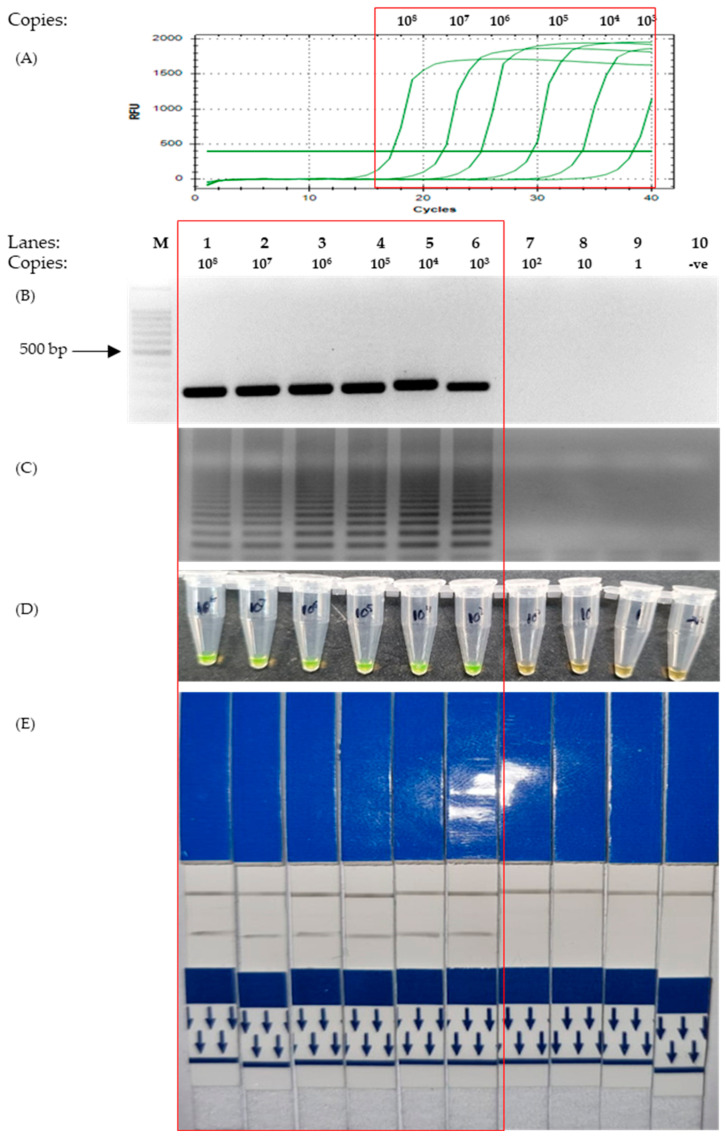
Sensitivity test to assess the detection limit of the SARS-CoV-2 membrane gene using different concentrations of positive control plasmids from 1 copy to 10^8^ copies (as indicated on the top of Figure 3B). The red box highlights the positive detection of SARS-CoV-2 M gene. (**A**) Quantitative PCR. (**B**) Agarose gel electrophoresis of PCR product under UV conditions and stained with EtBr. (**C**) Agarose gel electrophoresis of LAMP product under UV conditions with EtBr staining. (**D**) The corresponding LAMP products were stained with SYBR Green for colorimetric visualization. (**E**) The corresponding LAMP products are visualized on LFD. Lane M: 100 bp ladder, lane 1: 10^8^ copies, lane 2: 10^7^ copies, lane 3: 10^6^ copies, lane 4: 10^5^ copies, lane 5: 10^4^ copies, lane 6: 10^3^ copies, lane 7: 10^2^ copies, lane 8: 10 copies, lane 9: 1 copy, lane 10: non-template control.

The results for the sensitivity test of the M gene showed that the LAMP protocol was able to detect as little as 10^3^ copies of plasmids, as visualized with both UV and colorimetry by SYBR Green staining and LFD (Figure 3C–E). This is similar to that of the PCR tests, which were able to detect 10^3^ copies, as indicated by the graph and agarose gel photo in Figure 3A,B.

**Figure 4:**
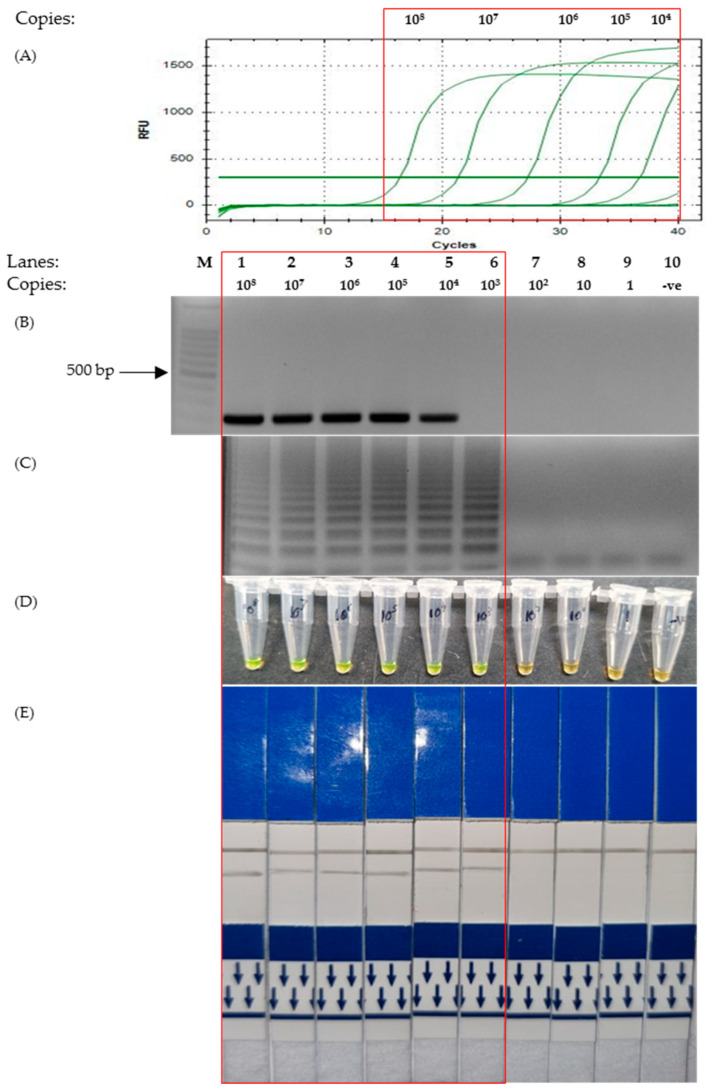
Comparison of the sensitivity test of the Envelope gene on various detection assays. The red box highlights the positive detection of SARS-CoV-2 E gene. Lane M: 100 bp ladder, lane 1: 10^8^ copies, lane 2: 10^7^ copies, lane 3: 10^6^ copies, lane 4: 10^5^ copies, lane 5: 10^4^ copies, lane 6: 10^3^ copies, lane 7: 10^2^ copies, lane 8: 10 copies, lane 9: 1 copy, lane 10: non-template control. (**A**) Quantitative PCR. (**B**) End-point PCR. (**C**) LAMP-UV. (**D**) LAMP-SYBR Green. (**E**) LAMP-LFD.

The sensitivity test showed that the detection limit of LAMP with regard to Envelope (E) gene primers was lower than that of the PCR. It can be observed that both qPCR and end-point PCR had a detection limit of 10^4^ copies (Figure 4A,B). However, as depicted in Figure 4C–E, the corresponding photos showed that the detection limit of LAMP was 10-fold lower at 10^3^ copies.

### 3.3. Specificity Test of LAMP-UV, LAMP-SYBR Green and LAMP-LFD

Prior to the development of LAMP, in silico screening was performed to design primers not only with conserved regions within SARS-CoV-2 variants but also with a low affinity toward the genes of closely related viral species. Specificity tests were successfully conducted on control plasmids with the gene inserts of various coronaviruses which had close genetic ties to SARS-CoV-2 and were available on hand, SARS-CoV-1, MERS-CoV and IBV.

**Figure 5:**
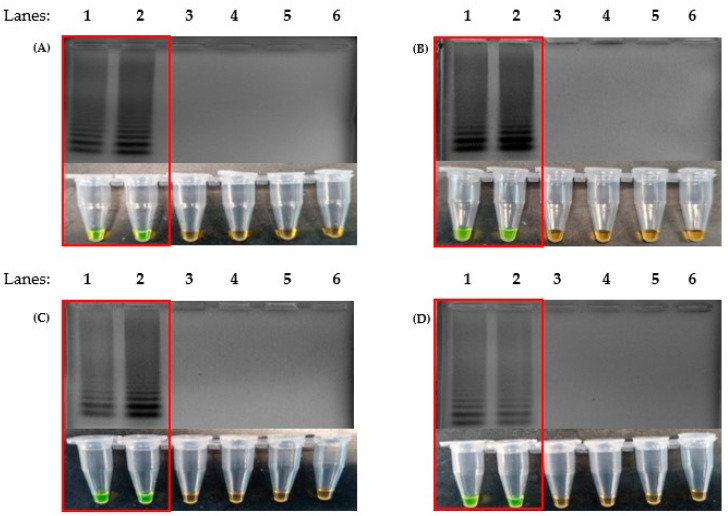
Specificity test to detect only the presence of SARS-CoV-2 genes without false positives, using the newly designed primers, N1 (**A**), N2 (**B**), M (**C**) and E (**D**). The red box highlights the positive detection. (Top) Agarose gel electrophoresis of the LAMP product under UV conditions and stained with ethidium bromide. (Bottom) The corresponding LAMP products were stained with SYBR Green for colorimetric visualization. Lane 1: 100 bp DNA ladder, lane 2: SARS-CoV-2 control plasmid, lane 3: SARS-CoV-2 synthetic RNA, lane 4: SARS-CoV-1 recombinant plasmid, lane 5: MERS-CoV recombinant plasmid, lane 6: cDNA of IBV, lane 7: non-template control.

Positive results indicated by ladder-like bands were observed only against the plasmids containing genes of SARS-CoV-2 (Figure 5). The DNA samples with genes of other coronaviruses produced negative results, as shown by the absence of ladder-like bands. Thus, the results from this specificity test for each set of LAMP primers implied that it was specific only to SARS-CoV-2.

### 3.4. Visualization of Multiplexed LAMP on a Single Strip of LFD

**Figure 6:**
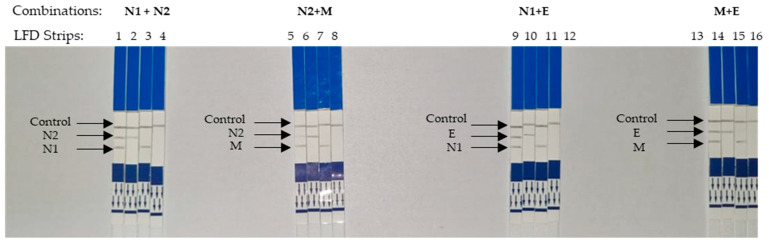
Visualization of multiplexed LAMP on LFD strips. LFD strips 1–4 are the results of multiplexed LAMP using primers N1 and N2. (Template used in strip 1: Control plasmid contained both N1 and N2 regions, strip 2: regions of N1 only, strip 3: regions of N2 only, strip 4: NTC). LFD strips 5–8 were multiplexed by LAMP using N1 and M primers. (Template used in strip 5: Control plasmid contained both N1 and M regions, strip 6: regions of N1 only, strip 7: regions of N2 only, strip 8: NTC). LFD strips 9–12 were multiplexed by LAMP using N2 and E primers. (Template used in strip 9: Control plasmid contained both N2 and E regions, strip 10: regions of E only, strip 11: regions of N2 only, strip 12: NTC). LFD strips 13–16 were multiplexed by LAMP using M and E primers. (Template used in strip 13: Control plasmid contained both M and E regions, strip 14: regions of E only, strip 15: regions of M only, strip 16: NTC).

Four different combinations were made from the designed primers (Figure 6). This was because the differentiating labels were only digoxigenin (N1 and E) and fluorescein (N2 and M). Therefore, primers with the same labels could not be used in combination. As observed, in a multiplexed reaction, two test lines were observed on a single LFD.

## 4. Discussion

Due to the expensive, time-consuming, and tedious nature of RT-PCR tests, healthcare professionals are opting for the less reliable but fast antigen-based rapid detection tests. Therefore, a simple and fast yet accurate detection method is needed for the detection of SARS-CoV-2 at early stages of infection. Taking this into consideration, a multiplexed LAMP-based approach was optimized as fast but also specific and sensitive.

The genes selected for this study were the N, M and E genes (Table 1). This was due to the high read coverage among coronavirus genes when RNA was sequenced from cultured tissues infected with HCoV-299E coronavirus [34]. Others reported LAMP-based SARS-CoV-2 detection using N, RdRp, S, ORF1ab, ORF8 and E genes [35,36,37,38,39,40,41]. In addition to that, through GISAID, we obtained genomic sequences for screening with the conserved regions within these genes. This was to ensure that the primers designed were able to detect SARS-CoV-2 across all variants. Therefore, false negative diagnoses were avoided. It was well described that to avoid false negatives, amplicons should be selected from conserved regions or multiple regions at the same time [42]. This was especially difficult as the viral genome is constantly mutating. In addition, even the primers from the gold standard, RT-PCR, were found to produce false negatives [8]. This was due to the ever so rapid occurrence of mutations at the common commercially used primer binding site.

A wide range of genomic sequences of SARS-CoV-2 was downloaded from the GISAID database (Appendix A Table A1), along with the reference genome from GenBank (NC_045512.2), as mentioned in Section 2.1. Using multiple alignment tools, we managed to obtain two conserved regions in the N gene and a region from the M and E genes, respectively, ranging from 200 to 220 bp in length. Six primers were designed for each set of primers, a pair for the inner and outer primers, respectively, as well as a pair of loop primers. Loop primers were found to increase the amplification speed of LAMP [19,30,43] as well as increase its specificity [44].

The LAMP protocol described In this study was able to detect SARS-CoV-2 control plasmids and synthetic RNA in as little as 10 min (Figure 1). With the use of *Bst,* 3.0 DNA polymerase (NEB), the time required for a one-step RT-LAMP reaction was reduced. This is due to the high reverse transcription activity of *Bst* 3.0 DNA polymerase: a single enzyme reaction could be performed using a single temperature (65 °C) [45]. Therefore, simple apparatus could be used to conduct the test (for example, a water bath or heat block). For optimum sensitivity, 30 min of incubation is ideal. Other LAMP-based detection methods have been described using various temperatures in the range of 60–65 °C with an extensive incubation time of up to 60 min. Two-step RT-LAMP protocols, however, take away the rapidness, thus making it unsuitable for point-of-care tests.

Plasmid DNA and synthetic RNA were used for the in vitro testing in the development of this multiplexed RT-LAMP-based LFD. The approach to amplify the genes using the outer primers was subsequently cloned for use as a template for the test, as previously described by others [30,46]. This provided a more accurate quantitative approach compared to repeated RNA extraction from SARS-CoV-2 virions. Furthermore, repeated exposure to SARS-CoV-2 brought a risk of infection during the experimentations.

The colorimetry LAMP visualization method used in this study was the addition of SYBR Green. Color changes were observed to indicate positive (green) and negative (orange) results (Figure 1A Bottom). However, as seen in lane 6 (Figure 3D and Figure 4D, respectively), the color changes we not as vivid. SYBR Green is a DNA intercalating dye with double-stranded DNA and showed color changes from orange to green [47,48]. This method is rapid in the sense that positive detection was observed with the naked eye without the need for agarose gel electrophoresis. However, at a low copy number of templates, the intermediate colors varied from individual observers as observed by others [49,50]. Therefore, colorimetric visualization is not the best option for LAMP. Other colorimetry dyes were documented for LAMP visualizations with varying success rates. This included phenol red [51,52], leuco crystal violet [53], calcein [54], and hydroxy-naphthol blue [55].

Another approach to minimize errors from calorimetry dyes is through the use of a lateral flow dipstick (LFD) in combination with the RT-LAMP, as described in this study. These clear distinct lines on LFD indicate the successful amplification of the N1, N2, M and E genes by LAMP. We used a carbon nanoparticle-based LFD with two distinct test lines targeting Biotin-fluorescein and Biotin–DIG complexes, respectively. The LFD presents tremendous prospects for point-of-care testing because it is straightforward, rapid and visual [56,57]. The genes amplified via LAMP with the primers listed in Table 1 exponentially increased the number of amplicons carrying Biotin-FAM or Biotin-DIG. Through capillary actions, the amplicons migrated through the LFD, where they bound to anti-biotin antibodies bonded with carbon nanoparticles, which were then mobilized [58]. Migrating amplicons carrying the carbon nanoparticle were immobilized at the test lines (coated with neutravidin or anti-DIG antibody). Thus, leaving the black lines observed on the test lines, a control line was formed due to the excess amplicons and/or biotin-labeled primers (no LAMP reaction) that were immobilized at the control line by unspecific antibodies. The use of RT-LAMP coupled with LFD reduced the need for potentially harmful carcinogens (for example, ethidium bromide stain in agarose gel electrophoresis), increased the accuracy (eliminates the use of colorimetric dyes), and even the use of expensive machinery (real-time PCR machine) [59,60,61].

The detection limit using N1, N2 and M primer sets was equal to that of PCR detection (Figure 2 and Figure 3), with the exception of primers for the E gene (Figure 4). This was because the sensitivity test for the E gene indicated that LAMP was more sensitive than the PCR. Furthermore, our sensitivity test was conducted with just 30 min of incubation in an isothermal condition (65 °C). With regard to LAMP, several publications were reportedly able to detect lower concentrations of the template material but required more time or the conduction of the reverse transcription process separately [62,63,64,65]. We used the isothermal enzyme *Bst* 3.0 DNA polymerase (NEB) for both reverse transcription and LAMP. The *Bst* 3.0 DNA polymerase has a dual activity of reverse transcriptase and polymerase in a single temperature incubation [32]. The use of this single enzyme here was efficient and made the one-step RT-LAMP more economical.

It is also important to consider the fact that the detection of low copies of SARS-CoV-2 did not indicate that a person was currently infected with COVID-19 [66]. A higher viral load was required for the body to show symptoms and severity increase with viral load [67]. It has previously been reported that positive qPCR results were observed weeks after infection, the majority of which had a high C_t_ value, which was an indication of a low viral load [68,69,70,71].

For the specificity test, the designed primers were indeed specific only toward the intended targets. Only the SARS-CoV-2 positive control plasmids and synthetic RNA revealed ladder-like bands, and none of the negative controls (SARS-CoV-1, MERS-CoV and IBV) were amplified (Figure 5). However, the control plasmids used in this study were limited due to the difficulty in procuring the genetic material of infectious pathogens. A more comprehensive screening was needed to further validate the results. Therefore, the in silico screening conducted earlier during primer design played an important role in the specificity of these primers.

The detection of two genes with two test lines on a single LFD is extremely relevant in diagnosis as this reduces the chances of false negatives (Figure 6). LFD is useful in multiplex LAMP (mLAMP) reactions as the amplification products could not be differentiated when using gel electrophoresis or visualized with SYBR Green. Therefore, a binary (positive or negative) interpretation of results may be flawed when it comes to multiplexed reactions. The different labels modified on the inner primers play an important role in LFD detection. A single tube assay along with the LFD is convenient for diagnosing COVID-19. Typically, two genes are required to further increase the specificity by avoiding false negatives. This is especially useful as the risk of mutation at one of the primer binding sites is ever-present. Others have described multiplexed molecular amplification when diagnosing SARS-CoV-2, notably using RT-PCR. In RT-PCR, these amplicons could be differentiated using different labels on the probes used. A multiplexed RT-PCR-based protocol [72] was recommended by WHO, which utilized the RdRp and E genes. This highly accurate multiplex RT-PCR approach, which has been utilized in many commercial kits, is still time-consuming and expensive.

While we acknowledge the need for clinical testing, our data show that the RT-LAMP-LFD protocol described here was accurate and rapid. Efforts are currently ongoing to utilize clinical samples to provide a more comprehensive examination of the RT-LAMP-LFD protocol developed from this study. Ongoing and not yet presented results on limited positive samples on hand revealed great promises. Due to varying geographical outbreaks, it was not possible to collect clinical samples of all the variants. Therefore, we decided it would be of interest to other researchers to release LAMP primers and protocols. Therefore, they could begin to test the samples available to them.

To further validate the protocol, trials using samples collected from swabs or saliva could be encouraged. Trials using crude saliva could generate high usefulness in point-of-care settings, preferably with variability in the variants. The convenience of direct testing from crude samples (saliva [73,74] and nasopharyngeal swabs [75]) was documented as before. Additionally, *Bst* 3.0 DNA polymerase was robust and capable of sustaining its activities in the presence of inhibitors [76]. This was especially useful for saliva and other samples which are known to carry amplification inhibitors [77]. This would ensure that the RT-LAMP-LFD was useful in a clinical setting.

## 5. Conclusions

The current protocol that we have developed is indeed rapid, sensitive, and specific in regard to the study procedures. Therefore, the development of a multiplexed RT-LAMP-LFD reaction on crude VTM samples would be suitable for point-of-care diagnosis of COVID-19 in diagnostic laboratories as well as in private homes.

## Figures and Tables

**Table 1:** The four sets of primers were designed for different gene targets consisting of the inner, outer and loop primers, along with the size of the target amplicon. The “automatic judgement” feature and default parameters from Primer Explorer Version 5 (http://primerexplorer.jp/lampv5e/index.html, accessed on 3 January 2021) were used to design each set of primers.

Primer Set	Target Gene	Primer Name	Sequence (5′ → 3′)	Target Size (bp)
N1	Nucleocapsid	F3_N1	CCAGAATGGAGAACGCAGTG	202
		B3_N1	CCGTCACCACCACGAATT	
		FIP_N1	Biotin-AGCGGTGAACCAAGACGCAGTTTTGGCGCGATCAAAACAACG	
		BIP_N1	DIG-AATTCCCTCGAGGACAAGGCGTTTTAGCTCTTCGGTAGTAGCCAA	
		LF_N1	TTATTGGGTAAACCTTGGGGC	
		LB_N1	TTCCAATTAACACCAATAGCAGTCC	
N2	Nucleocapsid	F3_N2	AGATCACATTGGCACCCG	213
		B3_N2	CCATTGCCAGCCATTCTAGC	
		FIP_N2	Biotin-TGCTCCCTTCTGCGTAGAAGCTTTTCAATGCTGCAATCGTGCTAC	
		BIP_N2	FAM-GGCGGCAGTCAAGCCTCTTCTTTTCCTACTGCTGCCTGGAGTT	
		LF_N2	AGATCACATTGGCACCCG	
		LB_N2	CCATTGCCAGCCATTCTAGC	
M	Membrane	F3_M	TCTTCTCAACGTGCCACT	220
		B3_M	CTGAGTCACCTGCTACAC	
		FIP_M	Biotin-TACGAAGATGTCCACGAAGGATTTTTCAGACCGCTTCTAGAAAGT	
		BIP_M	FAM-GGACACCATCTAGGACGCTGTTTTTAATAAGAAAGCGTTCGTGATG	
		LF_M	CACAGCTCCGATTACGAGTTC	
		LB_M	TGACATCAAGGACCTGCCT	
E	Envelope	F3_E	TCATTCGTTTCGGAAGAGA	205
		B3_E	GAACTCTAGAAGAATTCAGA	
		FIP_E	Biotin-CGCAGTAAGGATGGCTAGTGTATTTTCAGGTACGTTAATAGTTAATAGCG	
		BIP_E	DIG-TCGATTGTGTGCGTACTGCTGTTTTTTTTTAACACGAGAGTAAACGT	
		LF_E	CTAGCAAGAATACCACGAAAGC	
		LB_E	CAATATTGTTAACGTGAGTCTTGTA	

## Data Availability

Not applicable.

## Appendix A

**Table A1:** Full list of genome sequences of SARS-CoV-2 collected from the GISAID database, together with the countries of origin of samples from which the sequences were derived.

No.	Accession Number	Country of Origin
1	EPI_ISL_416866	Malaysia
2	EPI_ISL_430441	Malaysia
3	EPI_ISL_455312	Malaysia
4	EPI_ISL_501182	Malaysia
5	EPI_ISL_719133	Malaysia
6	EPI_ISL_718138	Malaysia
7	EPI_ISL_718174	Malaysia
8	EPI_ISL_738086	Malaysia
9	EPI_ISL_807150	Malaysia
10	EPI_ISL_807153	Malaysia
11	EPI_ISL_936488	Malaysia
12	EPI_ISL_936495	Malaysia
13	EPI_ISL_615652	Denmark
14	EPI_ISL_616802	Denmark
15	EPI_ISL_641491	Denmark
16	EPI_ISL_581117	United Kingdom
17	EPI_ISL_601443	United Kingdom
18	EPI_ISL_678386	Australia
19	EPI_ISL_728189	Singapore
20	EPI_ISL_733573	Hong Kong
21	EPI_ISL_739662	Canada
22	EPI_ISL_745260	Canada
23	EPI_ISL_755593	USA
24	EPI_ISL_755594	USA
25	EPI_ISL_755595	USA
26	EPI_ISL_755627	New Zealand
27	EPI_ISL_763074	Brazil
28	EPI_ISL_794625	New Zealand
29	EPI_ISL_803963	Singapore
30	EPI_ISL_842652	Argentina
31	EPI_ISL_843071	United Kingdom
32	EPI_ISL_845923	United Kingdom
33	EPI_ISL_846595	United Kingdom
34	EPI_ISL_849760	Australia
35	EPI_ISL_852526	United Kingdom
36	EPI_ISL_678594	South Africa
37	EPI_ISL_978596	South Africa
38	EPI_ISL_678597	South Africa
39	EPI_ISL_745169	South Africa
40	EPI_ISL_762992	South Korea
41	EPI_ISL_770472	Botswana
42	EPI_ISL_825398	Japan
43	EPI_ISL_825489	South Africa
44	EPI_ISL_843196	New Zealand
45	EPI_ISL_852547	United Kingdom
46	EPI_ISL_855369	France
47	EPI_ISL_855514	Kenya
48	EPI_ISL_1562503	USA
49	EPI_ISL_2550714	Malaysia
50	EPI_ISL_2803686	Zambia
51	EPI_ISL_2815331	Malaysia
52	EPI_ISL_2839566	Australia
53	EPI_ISL_2854187	Malaysia
54	EPI_ISL_2868394	Botswana
55	EPI_ISL_2876397	South Africa
56	EPI_ISL_2924057	Malaysia
57	EPI_ISL_2931921	Malaysia
58	EPI_ISL_2984856	South Africa
59	EPI_ISL_3019329	India
60	EPI_ISL_3049843	Kenya
61	EPI_ISL_3050795	Australia
62	EPI_ISL_3060617	India
63	EPI_ISL_3066408	India
64	EPI_ISL_3066431	India
65	EPI_ISL_3066449	India
66	EPI_ISL_3067537	USA
67	EPI_ISL_3071976	USA
68	EPI_ISL_833366	Japan
69	EPI_ISL_1250700	New Zealand
70	EPI_ISL_1416322	Australia
71	EPI_ISL_1428640	Japan
72	EPI_ISL_1543939	Singapore
73	EPI_ISL_1931621	Japan
74	EPI_ISL_2349709	Singapore
75	EPI_ISL_2769807	Japan
76	EPI_ISL_2933406	France
77	EPI_ISL_2956430	Germany
78	EPI_ISL_2988020	Turkiye
79	EPI_ISL_3033191	USA
80	EPI_ISL_3043979	Germany
81	EPI_ISL_3050309	Brazil
82	EPI_ISL_3050508	Brazil
83	EPI_ISL_3050610	Brazil
84	EPI_ISL_3072221	Brazil
85	EPI_ISL_3072616	Brazil
86	EPI_ISL_3087264	Belgium
87	EPI_ISL_3089659	Canada
88	EPI_ISL_416036	Brazil
89	EPI_ISL_431180	Fujian
90	EPI_ISL_445380	Thailand
91	EPI_ISL_490026	Australia
92	EPI_ISL_508266	India
93	EPI_ISL_522491	South Korea
94	EPI_ISL_579320	New Zealand
95	EPI_ISL_591450	Japan
96	EPI_ISL_630998	United Kingdom
97	EPI_ISL_640129	South Africa
98	EPI_ISL_640130	South Africa
99	EPI_ISL_672711	Brazil
100	EPI_ISL_690818	Japan
101	EPI_ISL_728187	Singapore
102	EPI_ISL_732179	Portugal
103	EPI_ISL_733300	Russia
104	EPI_ISL_746686	Chile
105	EPI_ISL_779245	Japan
106	EPI_ISL_779617	Australia
107	EPI_ISL_801402	Brazil
108	EPI_ISL_850198	South Korea
109	EPI_ISL_875048	United Kingdom
110	EPI_ISL_877765	Italy
111	EPI_ISL_901605	Japan
112	EPI_ISL_920984	Northern Ireland
113	EPI_ISL_941896	Portugal
114	EPI_ISL_985178	Brazil
115	EPI_ISL_1004317	Switzerland
116	EPI_ISL_648527	USA
117	EPI_ISL_707800	New Zealand
118	EPI_ISL_717710	Australia
119	EPI_ISL_755638	New Zealand
120	EPI_ISL_768628	Singapore
121	EPI_ISL_779199	Japan
122	EPI_ISL_818613	Denmark
123	EPI_ISL_846181	United Kingdom
124	EPI_ISL_857314	Taiwan
125	EPI_ISL_860112	Japan
126	EPI_ISL_872584	Australia
127	EPI_ISL_873881	United Kingdom
128	EPI_ISL_904760	Aruba
129	EPI_ISL_905242	Aruba
130	EPI_ISL_956331	Taiwan
131	EPI_ISL_967766	USA
132	EPI_ISL_972791	Denmark
133	EPI_ISL_982043	USA
134	EPI_ISL_984780	USA
135	EPI_ISL_985140	USA
136	EPI_ISL_762449	United Kingdom
137	EPI_ISL_906277	Nigeria
138	EPI_ISL_944748	Australia
139	EPI_ISL_995301	Singapore
140	EPI_ISL_1168766	USA
141	EPI_ISL_1168768	USA
142	EPI_ISL_1173226	Nigeria
143	EPI_ISL_1583653	Brazil
144	EPI_ISL_1896666	Denmark
145	EPI_ISL_1914650	Singapore
146	EPI_ISL_2155777	Philippines
147	EPI_ISL_2242809	Nigeria
148	EPI_ISL_2385974	Australia
149	EPI_ISL_2535627	Malaysia
150	EPI_ISL_3031386	Kenya
151	EPI_ISL_3063476	Turkiye
152	EPI_ISL_3089260	USA
153	EPI_ISL_861280	USA
154	EPI_ISL_896394	USA
155	EPI_ISL_1158385	USA
156	EPI_ISL_1698346	United Kingdom
157	EPI_ISL_1699692	United Kingdom
158	EPI_ISL_1721838	Germany
159	EPI_ISL_1994447	USA
160	EPI_ISL_2254415	USA
161	EPI_ISL_2967806	Spain
162	EPI_ISL_3032634	Turkiye
163	EPI_ISL_1360328	India
164	EPI_ISL_1442952	Singapore
165	EPI_ISL_1547802	India
166	EPI_ISL_1623010	Rep. Ireland
167	EPI_ISL_1647348	South Korea
168	EPI_ISL_1663320	India
169	EPI_ISL_1847409	Germany
170	EPI_ISL_2710315	South Africa
171	EPI_ISL_2762283	Germany
172	EPI_ISL_2882750	USA
173	EPI_ISL_1111128	Peru
174	EPI_ISL_1111321	Peru
175	EPI_ISL_1111341	Peru
176	EPI_ISL_1445272	Brazil
177	EPI_ISL_1477056	Spain
178	EPI_ISL_1494722	Australia
179	EPI_ISL_2492441	Mexico
180	EPI_ISL_2508552	Chile
181	EPI_ISL_2837340	USA
182	EPI_ISL_2876943	South Africa
